# Inhaled corticosteroids in COPD and the risk for coronary heart disease: a nationwide cohort study

**DOI:** 10.1038/s41598-020-74854-8

**Published:** 2020-11-04

**Authors:** Jiyoung Shin, Hee-Young Yoon, Yu Min Lee, Eunhee Ha, Jin Hwa Lee

**Affiliations:** 1 Department of Occupational and Environmental Medicine, College of Medicine, Ewha Womans University, Seoul, Republic of Korea; 2 Division of Pulmonary and Critical Care Medicine, Department of Internal Medicine, College of Medicine, Ewha Womans University, 25 Magokdong-ro 2-gil Gangseo-gu, Seoul, 07804 Republic of Korea

**Keywords:** Cardiovascular diseases, Disease prevention, Chronic obstructive pulmonary disease, Diseases, Medical research, Risk factors

## Abstract

Inhaled corticosteroids (ICS) might lower the risk of coronary heart disease (CHD) in patients with chronic obstructive pulmonary disease (COPD). This study aimed to assess the association of ICS with the development of CHD in COPD patients by using data from the Korean Nationwide study. Patients who were newly diagnosed with COPD between 2004 and 2013 and who were not diagnosed with coronary heart disease before their diagnosis of COPD were included. Exposure of ICS was incorporated into multivariable Cox regression models using time-dependent methods. To accurately estimate ICS-exposure accumulation, a washout period of 2 years from 2002 to 2003 was applied. Among a total of 4,400 newly diagnosed COPD patients, 771 patients were diagnosed as CHD incident cases during a median follow-up of one year (interquartile range 0.1–2.9). The cumulative dose of ICS was associated with a reduced risk of CHD (adjusted hazard ratio [aHR], 0.68; 95% confidence interval [CI], 0.52–0.89). When the cumulative exposure dose of ICS was divided into quartiles, the aHR for CHD incidence was 0.70 (95% CI, 0.55–0.88) in the highest quartile ICS dose use. The effect of ICS on reducing CHD incidence was pronounced in adults over 55 years, men under 55 years, and former smokers. Our findings demonstrate the role of ICS for the prevention of CHD in COPD patients without a history of CHD. Further research is needed to determine whether a certain amount of ICS exposure in COPD patients is protective against CHD.

## Introduction

Chronic obstructive pulmonary disease (COPD) is a common lung disease characterized by persistent airflow limitation and chronic respiratory symptoms due to exposure to environmental factors such as smoking in genetically susceptible individuals^1^. Pharmacological treatment of COPD aims to alleviate respiratory symptoms and prevent acute exacerbation (AE). Inhalation medications including long-acting muscarinic antagonists (LAMA), long-acting beta-2 agonists (LABA), and inhaled corticosteroids (ICS) are now used as the first line therapy according to the clinical status of each patient^1,2^. ICS is generally combined with LABA in a single device. These combined inhalers are recommended as initial therapy for selected COPD patients with an asthmatic component, severe respiratory symptoms, or frequent AEs; such patients have been previously identified to reap high benefits from ICS therapy^3–5^. However, survival gain from ICS is not demonstrated in several randomized controlled trials (RCTs)^6,7^.

Cardiovascular disease is a common comorbidity in COPD and a major contributor to COPD mortality^8,9^. Whether the use of ICS can influence mortality caused by cardiovascular disease is still debatable. Two observational studies reported that ICS reduced the risk of acute myocardial infarction in patients with asthma and COPD^10,11^, while a large-scale RCT, SUMMIT (Study to Understand Mortality and Morbidity in COPD), of 16,590 patients with moderate COPD at high risk for cardiovascular events failed to demonstrate the same effects of ICS on mortality or cardiovascular outcomes^7,12^. However, as the SUMMIT study had enrolled patients who were already on ICS/LABA combination or its ingredients, it is difficult to distinguish between results that are because of the direct effect of new medications or the residual effect of prior medications. In Korea, the National Health Insurance Service (NHIS) manages the National Sample Cohort including about a million representative subjects. Using these data, it is possible to investigate disease incidence and related risk factors in a large-scale national population. Therefore, in this study, our aim was to assess the effect of ICS on coronary heart disease (CHD) in COPD patients.

## Methods

### Data source

The NHIS of South Korea, a health insurance system with universal coverage, constructed the National Health Information Database (NHID) that contains personal information, demographic data, and medical treatment information on almost all Koreans (> 97%) from 2000. In 2015, the NHIS released the data of the Korean National Health Insurance Service-National Sample Cohort (NHIS-NSC), which is a representative, population-based cohort proportionally stratified by age, sex, and income levels^13^. A total of 1,125,691 subjects were enrolled the cohort and followed-up from January 1, 2002 until December 31, 2013.

The NHIS-NSC is a semi-dynamically constructed cohort database, and during the follow-up period, the cohort was refreshed annually by adding a representative sample of newborns, sampled across 82 strata (two for sex, combined with 41 for parents’ income levels) using a 2.2% sampling rate. The NHIS-NSC comprises databases based on a participant’s insurance eligibility and medical treatments that contain information about participants’ medical bills claimed by medical service providers, medical care institutions, and general health examinations. The general health examination database comprises information about nationwide health examination conducted by the NHIS in 2002–2013 and contains major health examination results pertaining to lifestyles and behaviors obtained from questionnaires.

### Study population and case definition

We obtained the study population data of the years 2002–2013 from the NHIS-NSC. COPD patients were defined as persons who were diagnosed with COPD based on the Korean classification of diseases, 6th revision (KCD-6) code at least twice a year from January 1, 2004 to December 31, 2013. We identified the diagnosis data from patient’s medical treatment based on KCD-6, which is the modified version of the international classification of disease, 10th revision (ICD-10). We set the criteria for COPD patients based on the KCD-6 code including J42.x to J44.x except J430 (Supplement Table 1). To analyze the effect of ICS exposure, only patients who were newly diagnosed with COPD and were prescribed an inhalant afterwards were included in the study. Therefore, washout periods for a total of two years were established; this excluded patients who had been diagnosed with COPD or were prescribed inhalers between the years 2002 and 2003.

**Table 1:** Characteristics of the study subjects^a^.

Variables	Total (n = 4,400)	CHD (n = 771)	Non-CHD (n = 3,629)	*p* value^b^
**Follow up duration (years)**	3.3 ± 2.7	1.8 ± 2.1	3.6 ± 2.7	< 0.001
	2.7 [0.9, 5.2]	1.0 [0.1, 2.9]	3.0 [1.3, 5.7]	
**Age (years)**	59.1 ± 11.5	63.7 ± 9.9	58.1 ± 11.6	< 0.001
< 55	1,502 (34.1%)	139 (18.0%)	1,363 (37.6%)	
≥ 55	2,898 (65.9%)	632 (82.0%)	2,266 (62.4%)	
**Men**	2,468 (56.1%)	454 (58.9%)	2,014 (55.5%)	0.08
**Smoking status**				0.06
Never smokers	2,615 (59.4%)	461 (59.8%)	2,154 (59.4%)	
Former smokers	773 (17.6%)	153 (19.8%)	620 (17.1%)	
Current smokers	1,012 (23.0%)	157 (20.4%)	855 (23.6%)	
**Body mass index (kg/m**^**2**^**)**	23.4 ± 3.5	23.4 ± 3.8	23.4 ± 3.5	0.76
**CCI**	1.3 ± 1.3	1.9 ± 1.7	1.2 ± 1.2	< 0.001
**Household income levels**				0.08
0–20%	837 (19.0%)	160 (20.8%)	677 (18.7%)	
20–40%	549 (12.5%)	86 (11.2%)	463 (12.8%)	
40–60%	706 (16.1%)	103 (13.4%)	603 (16.6%)	
60–80%	894 (20.3%)	159 (20.6%)	735 (20.3%)	
80–100%	1,414 (32.1%)	263 (34.1%)	1,151 (31.7%)	
**Medication**				
ICS	2,683 (61.0%)	453(58.8%)	2,230 (61.5%)	0.16
ICS/LABA	1,340 (30.5%)	199 (25.8%)	1,141 (31.4%)	0.002
LABA	12 (0.3%)	4 (0.5%)	8 (0.2%)	0.15
LAMA	459 (10.4%)	66 (8.6%)	393 (10.8%)	0.06
SABA	1,481 (33.7%)	286 (37.1%)	1,195 (32.9%)	0.03
SABA/SAMA	26 (0.6%)	7 (0.9%)	19 (0.5%)	0.21
SAMA	369 (8.4%)	100 (13.0%)	269 (7.4%)	< 0.001
**Asthma status **^**c**^				0.17
No	724 (16.5%)	114 (14.8%)	610 (16.8%)	
Yes	3,676 (83.6%)	657 (85.2%)	3,019 (83.2%)	

CCI, Charlson comorbidity index; CHD, coronary heart disease; ICS, inhaled corticosteroids; LABA, long-acting beta-2 agonists; LAMA, long-acting muscarinic antagonists; SABA, short-acting beta-2 agonists; SAMA, short-acting muscarinic antagonists.

^a^Data are n (%), or mean ± SD, or median [interquartile range].

^b^*p* value for chi square test or *t*-test.

^c^In the year of inhaler initiation

The primary outcome of our study—CHD—was defined according to KCD-6 code and procedure code within the NHIS-NSC medical treatment database. Consistent with the American Heart Association, KCD-6 codes of I20 to I25 for CHD and procedure codes were used (Supplement Tables 1, 2)^14^. A CHD event was defined as a case in which one or more of the following three conditions were satisfied: (i) at least one hospitalization based on the KCD-6 code for CHD; (ii) At least two visits to the outpatient department based on the KCD-6 codes for CHD; and (iii) At least one revascularization based on the procedure code defined as coronary artery bypass grafting or percutaneous coronary intervention^15–17^. Patients diagnosed with the CHD event before inhaler use were excluded.

**Table 2:** Associations between COPD patients with or without the use of ICS and the risk of coronary heart disease^a^.

	Crude Hazard ratio (95% CI)	*p* value	Adjusted Hazard ratio (95% CI)	*p* value
Total	0.97 (0.83–1.14)	0.71	1.13 (0.96–1.32)	0.14
Adults ≥ 55 years^b^	1.07 (0.90–1.27)	0.44	1.08 (0.91–1.28)	0.38
Males < 55 years^c^	0.74 (0.44–1.24)	0.74	0.76 (0.45–1.29)	0.31
Females < 55 year ^c^	1.62 (0.88–3.00)	0.12	1.55 (0.83–2.88)	0.17
Never smoker^d^	0.94 (0.77–1.14)	0.51	1.12 (0.91–1.37)	0.30
Former smoker^d^	0.81 (0.57–1.15)	0.24	0.87 (0.61–1.26)	0.46
Current smoker^d^	1.27 (0.88–1.84)	0.20	1.42 (0.98–2.07)	0.06

COPD, chronic obstructive; ICS, inhaled corticosteroids; CI, confidence interval.

^a^ICS usages are analyzed as time-dependent covariates.

^b^Adjusted HRs were adjusted for age, sex, body mass index, household income level, Charlson comorbidity index, and smoking status.

^c^Adjusted HRs were adjusted for body mass index, household income level, Charlson comorbidity index, and smoking status.

^d^Adjusted HRs were adjusted for age, sex, body mass index, household income level, and Charlson comorbidity index.

We divided the study subjects into three groups based on age and sex: Patients aged ≥ 55 years, men < 55 years, and women < 55 years. We did this because numerous studies have suggested that estrogen protects the heart, and thus, the risk of cardiovascular disease is much lower in premenopausal women than in men^18^. After menopause, there is no difference in the incidence of cardiovascular disease between men and women^19^. Given that 97.6% of Korean women are menopausal at the age of 55 years^20^, we divided the study subjects using the threshold of 55 years.

### Exposure to inhalers

A patient who was prescribed inhalation respiratory medicine at least twice a year after diagnosed COPD was defined as an inhaler user and included in the study. Patients who had prescription records of inhalers before a COPD diagnosis were excluded. We set this “at least twice a year” criteria to exclude the possible miscoding of data. The inhaled respiratory medicine included ICS (beclomethasone, budesonide, ciclesonide, flunisolide, fluticasone, or triamcinolone); short-acting beta-2 agonists (SABA; fenoterol, procaterol, salbutamol, or terbutaline); LABA (formoterol or salmeterol); short-acting muscarinic antagonists (SAMA; ipratropium); LAMA (tiotropium); and a combination of SABA/SAMA (ipratropium/salbutamol) or ICS/LABA (budesonide/formoterol, fluticasone/salmeterol).

ICS users were defined as those who were prescribed ICS at least twice a year after COPD diagnosis, and non-ICS users were defined as inhaler users who did not meet the criteria for ICS users. The index date from which the follow-up period began was identified as the point at which the first inhaler of the inhaler users was prescribed. The time before the use of ICS was reallocated to non-ICS user to overcome the immortal time bias. All subjects were followed-up to the earliest date of coronary heart disease occurrence, death, or the end of 2013.

The year of the first prescription of inhaled respiratory medicine was defined as the inhaler initiation year. The ICS dose was converted to fluticasone equivalents, and the cumulative dose of ICS was calculated by aggregating the total dose of medication prescribed to a specific patient follow-up period. The equivalent doses for ICS were 100 mg beclomethasone, 50 mg beclomethasone hydrofluoralkane, 80 mg budesonide, 200 mg triamcinolone, 32 mg ciclesonide, 50 mg fluticasone, and 200 mg flunisolide^21–23^.

### Statistical analysis

Demographic characteristics between the CHD and non-CHD groups were compared using the ${\upchi }^{2}$ test for categorical variables and the *t*-test for continuous variables. Among inhaler users with COPD, Kaplan–Meier curves were generated based on ICS use, sex, high/low dose of ICS (divided the distribution of the cumulative ICS dose into ≥ 75th and < 75th percentile of the total dose) and smoking status; the log-rank test was used out to compare the Kaplan–Meier curves.

A Cox proportional hazards model was used to calculate the adjusted hazard ratio (aHR) with 95% confidence interval (CI) to assess the association between time-dependent ICS use and CHD in COPD patients. We also set cumulative dose of ICS below the 25th percentile as the referent category and categorized dose exposure levels into quartiles with three cut-off points (25th, 50th, and 75th percentiles) to compare the results based on the lowest quartile of ICS dose. In the percentile analysis, cumulative dose of ICS in non-ICS users was considered as zero.

In terms of covariates, demographic covariates included: age, sex, body mass index (BMI) and household income level (5 strata). We also included smoking status^24^ and Charlson comorbidity index (CCI)^25^ as covariates. All covariates were CHD risk factors assessed during the national health examination in the year of CHD diagnosis. Covariates data for the non-CHD patients were collected from the latest record in the national health examination database.

We also performed stratified subgroup analyses by dividing the patients into subgroups for age and sex (adult aged ≥ 55 years, men < 55 years, and women < 55 years). Furthermore, study participants were stratified into three smoking categories based on self-reported smoking status: never smokers, former smokers, and current smokers.

All p-values were two-sided, and those < 0.05 were considered to indicate statistical significance. All statistical analyses were performed using SAS 9.4 software (SAS Institute, Cary, NC, USA).

### Ethics approval

This study was approved by the Institutional Review Board of Ewha Womans University Mokdong Hospital, Seoul, Republic of Korea (IRB Number: EUMC 2019-01-014).

## Results

### Subject enrollment

**Figure 1:**
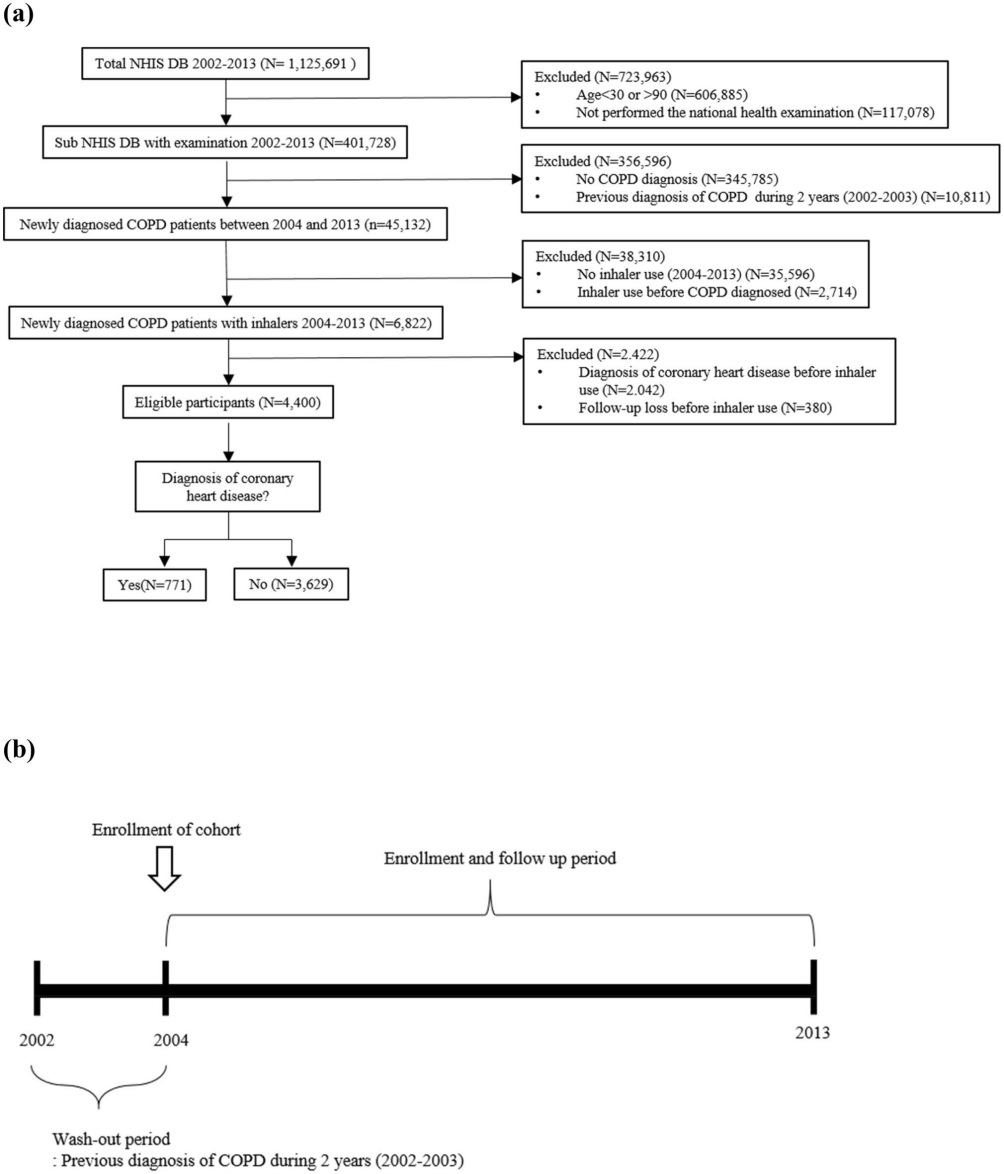
Study design (**a**) Flow diagram of the study design (**b**) Study design over time. NHIS DB, National Health Insurance Service database; COPD, chronic obstructive lung disease.

From the entire NHIS database between 2002 and 2013 (n = 1,125,691), subjects aged < 30 years (n = 606,885) and those who did not have a record of a national health examination (n = 117,078) were excluded. From the remaining subjects, those without a COPD diagnosis code (n = 345,785) or with a COPD diagnosis code during the washout period of 2002 and 2003 (n = 10,811) were excluded. Furthermore, subjects who did not have prescription records of inhalers (n = 35,596) or had prescription records of inhalers before a COPD diagnosis (n = 2,714) were also excluded. Finally, after excluding subjects with a CHD diagnosis code before inhaler prescription (n = 2,042) and follow-up loss before inhaler use (n = 380), 4,400 subjects were included in our study (Fig. 1).

### Baseline demographics

Of the 4,400 subjects, 56.1% were men with a mean age of 59.1 ± 11.5 years (Table 1). Among them, 771 (17.5%) were defined as cases with CHD. The subjects with CHD were older and had a higher CCI than those without CHD. Regarding the proportions of the inhalers, the use of ICS/LABA, SABA, and SAMA significantly differed between the CHD and non-CHD groups. We also evaluated the presence of asthma in the inhaler initiation year of each patient. This was ascertained based on the relevant KCD-6 codes (J45, J46) at least twice in the inhaler initiation year.

### Incidence of CHD

**Figure 2:**
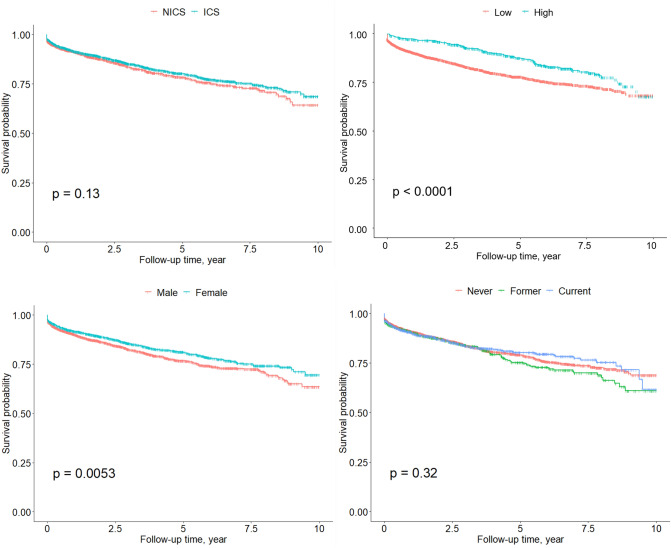
Kaplan Meier curves according to (**a**) use of ICS, (**b**) cumulative dose of ICS ^a^, (**c**) sex, and (**d**) smoking status among COPD patients with inhaler use. ^a^ High cumulative dose > 75% of total cumulative dose distribution (75 percentile of cumulative dose of ICS = 207,500 $\mathrm{\mu g}$ of fluticasone equivalent).

The Kaplan–Meier curves highly suggested non-proportional hazards regarding the association between ICS usage and CHD (*P* = 0.13, Fig. 2a), and between smoking status and CHD (*P* = 0.32, Fig. 2d). However, Kaplan–Meier curves exhibited significant differences in the development of CHD by high/low cumulative ICS dose usage (*P* < 0.001, Fig. 2b); and sex (*P* = 0.005, Fig. 2c).

### ICS usage and the risk of CHD

In the reference case, which classified ICS exposure as a time-dependent variable, the use of ICS was not associated with a lower risk of CHD in both crude and adjusted models (Table 2). However in analyzes based on the cumulative dose of ICS adjusted to covariates, analysis of the total group showed a decrease in CHD risk with increasing cumulative ICS doses (HR, 0.68, 95% CI, 0.52–0.89) (Table 3). In the subgroup analysis, adult over 55 years, men under 55 years, women under 55 years, and former smokers group showed significant association between the cumulative use of ICS and lower risk of CHD.

**Table 3:** Associations between cumulative^ab^ dose of ICS exposure and the risk of coronary heart disease in COPD patients.

	Crude Hazard ratio (95% CI)	*p* value	Adjusted Hazard ratio (95% CI)	*p* value
Total	0.68 (0.52–0.89)	0.005	0.68 (0.52–0.89)	0.004
Adults ≥ 55 years^c^	0.75 (0.58–0.99)	0.039	0.74 (0.56–0.97)	0.004
Males < 55 years^d^	0.29 (0.07–1.15)	0.078	0.66 (0.48–0.91)	0.011
Females < 55 years^d^	0.34 (0.07–1.76)	0.20	0.56 (0.32–1.00)	0.049
Never smoker^e^	0.67 (0.45–1.00)	0.052	0.73 (0.49–1.07)	0.11
Former smoker^e^	0.54 (0.30–0.97)	0.040	0.51 (0.28–0.92)	0.026
Current smoker^e^	0.81 (0.52–1.24)	0.32	0.81 (0.51–1.28)	0.36

COPD, chronic obstructive pulmonary disease; ICS, inhaled corticosteroids; CI, confidence interval.

^a^Measured as a continuous variable (grams).

^b^ICS usages are analyzed as time-dependent covariates.

^c^Adjusted HRs were adjusted for age, sex, body mass index, household income level, Charlson comorbidity index, and smoking status.

^d^Adjusted HRs were adjusted for body mass index, household income level, Charlson comorbidity index, and smoking status.

^e^Adjusted HRs were adjusted for age, sex, body mass index, household income level, and Charlson comorbidity index.

**Figure 3:**
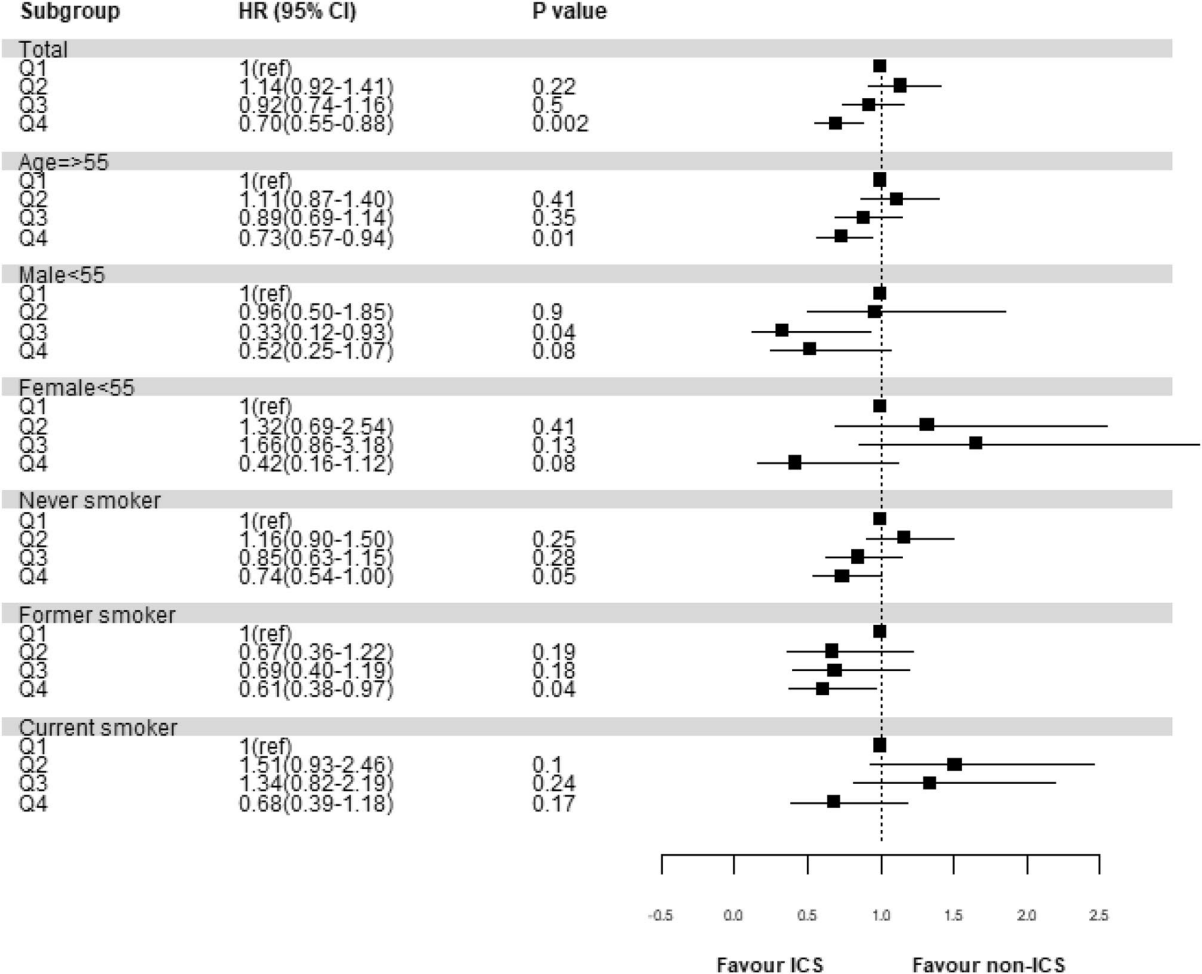
Associations between quartiles of ICS exposure and the risk of coronary heart disease^ab^. ^a^Models adjusted for age, sex, body mass index, household income level, Charlson comorbidity index, and smoking status. ^b^Quartile 1(< 30,000 $\mathrm{\mu g}$ of fluticasone equivalent), Quartile 2(30,000–73,125 $\mathrm{\mu g}$ of fluticasone equivalent), Quartile 3(73,125–207,500 $\mathrm{\mu g}$ of fluticasone equivalent), and Quartile 4(> 207,500 $\mathrm{\mu g}$ of fluticasone equivalent).

When the cumulative dose of ICS was divided into quartiles, including zero (= non-ICS group), the highest quartile of the cumulative dose of ICS showed a lower risk of CHD in the entire study population (HR, 0.70; 95% CI 0.55–0.88) and some subgroups. In particular, the three subgroups namely, adults over 55 years, men < 55 years and former smokers appear to have the greatest gain in CHD reduction through ICS use; whereas, women < 55 years and current smokers seemed to have relatively low benefit (Fig. 3).

## Discussion

Our study included subjects from the national insurance cohort to assess the association between the use of ICS and risk of CHD in COPD patients with inhalers. In the reference case, which classified ICS exposure as an ICS use or non-ICS use time-dependent variable, the use of ICS was not associated with a lower risk of CHD. Since COPD patients with very little ICS exposure were also classified as ICS users, it is highly likely that the results of the analysis by simply dividing time based on ICS use and non-ICS use may not accurately demonstrate the effect of ICS on CHD occurrence. On the other hand, as a result of three additional analyses on ICS exposure dose (continuous, high/low, and quartiles), high cumulative dose of ICS was associated with a reduction in CHD events. Its protective effect was prominent in adults over 55 years, men under 55 years, and former smokers.

Although the association between ICS use and CHD in COPD patient has been evaluated in several studies, the results are still controversial. Huiart et al. used the Canada Health Insurance databases and reported that COPD patients with a low dose of ICS (50–200 µg/day beclomethasone-equivalent) were associated with a lower risk of acute myocardial infarction (relative risk: 0.68; 95% CI, 0.47–0.99) than those without ICS; however, other ICS doses did not show a significant association^11^. The post hoc analysis of European Respiratory Society’s study on Chronic Obstructive Pulmonary Disease (EUROSCOP) including 1,175 smokers with mild COPD (FEV_1_: 50–100% predicted) revealed that patients with inhaled budesonide (800 μg/day) had lower incidence of ischemic cardiac events than those with placebo (relative risk: 0.58; 95% CI, 0.35–0.98) during the 3-year follow-up^26^. Conversely, post hoc analysis of 3-year data from the Towards a Revolution in COPD Health (TORCH) study revealed that patients who inhaled fluticasone propionate experienced similar rates of cardiovascular events, including ischemic cardiovascular events, as patients with placebo, with LABA alone, and with ICS/LABA^27^. In our study, the cumulative dose use of ICS was significantly related to the reduced risk of CHD. Unlike previous studies, because our study only included newly diagnosed COPD patients with initiation of ICS, we believe we have evaluated the effectiveness of solely ICS than other studies. We also investigated the incidence of CHD and ICS only in COPD patients who were not previously diagnosed with cardiovascular disease. Furthermore, in this study, ICS medication was analyzed as a time-dependent covariate in the Cox proportional-hazards model to eliminate immortal time bias which can generate an illusion of treatment effectiveness^28^.

Although the mechanism of ICS for protecting CHD remains unclear, it might be related to the reduction in AE-COPD by ICS use. Several studies have shown an association between AE-COPD and the risk of cardiovascular disease^29–31^. The SUMMIT study found that risk of cardiovascular disease was significantly increased after AE-COPD in 16,485 COPD patients with enhanced risk of cardiovascular disease^29^. Therefore, the control of AE should be focused on to reduce cardiovascular disease in COPD patients. In many studies, the use of ICS in combination with bronchodilators has been shown to prevent AE, reduce lung function decline, and improve the quality of life in COPD patients^4, 5^, particularly for patients with frequent exacerbations^32^. Therefore, ICS is recommended for patients with a history of AE^1^. These findings are in agreement with our results in that the use of ICS showed superior efficacy for reducing CHD in ICS users with a high cumulative dose. Considering that ICS is usually prescribed for frequent exacerbators or severe COPD patients, our subjects with COPD and ICS use were more likely to have a relatively high risk of CHD. Nevertheless, the higher was the ICS exposure, the greater was the effect of reducing CHD risk. Additionally, the improvement of hyperinflation and hypoxemia, which were predecessors of cardiac burden in severe COPD^33^, due to ICS use might be associated with reduction of CHD risk.

Another explanation for the mechanism of CHD prevention by ICS is that it may be related to a reduction in systemic inflammation by ICS. Past evidence has shown that cardiovascular disease is provoked by chronic inflammation^34^. Systemic inflammation recruits inflammatory cells and cytokines and contributes to all phases of atherosclerosis, which accounts for most of the acute complications of CHD^34^. The proinflammatory markers associated with cardiovascular risk include interlukin-6, tumor necrosis factor-alpha, and C-reactive protein^35–37^, which are suppressed by ICS in human studies^38^. These findings collectively support the association between ICS and CHD via controlling systemic inflammation.

In our study, current smokers had less cardio-protective effect from ICS than never and former smokers. Surya et al. reported that the responses to ICS in current smokers was less than those in former smokers in the SUMMIT trial. The effect of ICS/LABA on FEV_1_ improvement and reduction in AE were lower in current smokers than in former smokers^39^. Further prospective RCTs are needed to determine whether ICS has protective effects for COPD patients stratified by smoking status.

Our study has some limitations. First, as this was an observational study, potential confounders, including lung function and underlying disease, may contribute to the occurrence of CHD. However, we have not only adjusted for major risk factors for CHD, such as age, sex, and smoking status, but also adjusted for comorbidities using CCI, which is known to predict 10-year survival in patients with comorbidities. Second, because ICS is mainly used as a combination with other bronchodilators in COPD patients, our results might be inadequate to observe the effect of ICS alone. However, there were no significant differences in the use of LABA, LAMA, and SABA/SAMA between the CHD and non-CHD groups. Third, this is a study using a claim database, and there were no data on actual clinical information from laboratory tests, pulmonary function tests, and respiratory symptoms. Although we defined the diagnosis and outcome using structural definitions, the possibility of over-diagnosis or misdiagnosis could not be totally excluded because the diagnosis of COPD or CHD was not confirmed by accurate tests such as spirometry or coronary artery angiography. Fourth, despite the fact that our study subjects were COPD patients, the proportions of women and never smokers were relatively high. This may be due to the limitations of applying operational definitions without diagnosing COPD based on spirometry results. Another explanation is that this study excluded people who did not receive a national examination, which includes collecting information about smoking status, body mass index, and household income data. It is generally known that compared with non-smokers, smokers have lower interest in healthcare and therefore have lower rates of national examinations. Therefore, the proportion of smokers is likely to be relatively low; moreover, the proportion of male patients is likely to be relatively low because the smoking rate of Korean men is much higher than that of Korean women.

In conclusion, our study highlighted the role of high cumulative dose of ICS for the prevention of CHD in COPD patients without a history of CHD. Because the occurrence of CHD leads to a poor prognosis of COPD, it is crucial to reduce the risk of CHD. Particularly, given the fact that a cumulative dose of ICS was related to a reduction in CHD, controlling COPD may be important to prevent CHD. Considering the risk–benefit ratio of ICS, further prospective studies are necessary to determine the optimal dose and duration of ICS in COPD patients.

## Supplementary information

Supplementary Information
