# Supplementary material for: Inhaled corticosteroids in COPD and the risk for coronary heart disease: a nationwide cohort study
Source: Sci Rep. 2020 Nov 4;10:18973. doi: 10.1038/s41598-020-74854-8 (PMC7642419; doi:10.1038/s41598-020-74854-8)
Supplement: Supplementary file 1 — Supplementary Information [file 41598_2020_74854_MOESM1_ESM.docx]

**Inhaled Corticosteroids in COPD and the Risk for Coronary Heart Disease: A Nationwide Cohort Study**

Jiyoung Shin^1^*, Hee-Young Yoon^2^*, Yu Min Lee^1^, Eunhee Ha^1‡^, Jin Hwa Lee^2‡^

^1^ Department of Occupational and Environmental Medicine, College of Medicine, Ewha Womans University, Seoul, South Korea

^2^ Division of Pulmonary and Critical Care Medicine, Department of Internal Medicine, College of Medicine, Ewha Womans University, Seoul, South Korea

* These authors contributed equally to this work as a first author.

^‡^ These authors contributed equally to this work as a corresponding author.

**Correspondence**

Eunhee Ha

Department of Occupational and Environmental Medicine,

College of Medicine, Ewha Womans University, Seoul, Republic of Korea.

Tel: +82-2-2650-5757

Fax: +82-2-2653-1086

E-mail: eunheeha@ewha.ac.kr

Jin Hwa Lee

Division of Pulmonary and Critical Care Medicine, Department of Internal Medicine, College of Medicine, Ewha Womans University, Seoul, Republic of Korea

25 Magokdong-ro 2-gil Gangseo-gu, Seoul, 07804, Republic of Korea.

Tel: +82-2-6986-1631

E-mail: jinhwalee@ewha.ac.kr

Supplement table 1. Korean Classification of Diseases, 6^th^ revision (KCD-6) codes used for defining COPD and CHD.

| Disease | KCD-6 Code | Description |
| --- | --- | --- |
| Chronic obstructive lung disease (COPD) | J42 | Unspecified chronic bronchitis |
|  | J43 | Emphysema |
|  | J43.1 | Panlobular emphysema |
|  | J43.2 | Centrilobular emphysema |
|  | J43.8 | Other emphysema |
|  | J43.9 | Emphysema, unspecified |
|  | J44 | Other chronic obstructive pulmonary disease |
|  | J44.0 | Chronic obstructive pulmonary disease with acute lower respiratory infection |
|  | J44.1 | Chronic obstructive pulmonary disease with acute exacerbation, unspecified |
|  | J44.8 | Other specified chronic obstructive pulmonary disease |
|  | J44.9 | Chronic obstructive pulmonary disease, unspecified |
| Coronary heart disease (CHD) | I20 | Angina pectoris |
|  | I20.0 | Unstable angina |
|  | I20.1 | Angina pectoris with documented spasm |
|  | I20.8 | Other forms of angina pectoris |
|  | I20.9 | Angina pectoris, unspecified |
|  | I21 | Acute myocardial infarction |
|  | I21.0 | Acute transmural myocardial infarction of anterior wall |
|  | I21.1 | Acute transmural myocardial infarction of inferior wall |
|  | I21.2 | Acute transmural myocardial infarction of other sites |
|  | I21.3 | Acute transmural myocardial infarction of unspecified site |
|  | I21.4 | Acute subendocardial myocardial infarction |
|  | I21.9 | Acute myocardial infarction, unspecified |
|  | I22 | Subsequent myocardial infarction |
|  | I22.0 | Subsequent myocardial infarction of anterior wall |
|  | I22.1 | Subsequent myocardial infarction of inferior wall |
|  | I22.8 | Subsequent myocardial infarction of other sites |
|  | I22.9 | Subsequent myocardial infarction of unspecified site |
|  | I23 | Certain current complications following acute myocardial infarction |
|  | I23.0 | Haemopericardium as current complication following acute myocardial infarction |
|  | I23.1 | Atrial septal defect as current complication following acute myocardial infarction |
|  | I23.2 | Ventricular septal defect as current complication following acute myocardial infarction |
|  | I23.3 | Rupture of cardiac wall without haemopericardium as current complication following acutemyocardial infarction |
|  | I23.4 | Rupture of chordae tendineae as current complication following acute myocardial infarction |
|  | I23.5 | Rupture of papillary muscle as current complication following acute myocardial infarction |
|  | I23.6 | Thrombosis of atrium, auricular appendage, and ventricle as current complicationsfollowing acute myocardial infarction |
|  | I23.8 | Other current complications following acute myocardial infarction |
|  | I24 | Other acute ischaemic heart diseases |
|  | I24.0 | Coronary thrombosis not resulting in myocardial infarction |
|  | I24.1 | Dressler’s syndrome |
|  | I24.8 | Other forms of acute ischaemic heart disease |
|  | I24.9 | Acute ischaemic heart disease, unspecified |
|  | I25 | Chronic ischaemic heart disease |
|  | I25.0 | Atherosclerotic cardiovascular disease, so described |
|  | I25.1 | Atherosclerotic heart disease |
|  | I25.2 | Old myocardial infarction |
|  | I25.3 | Aneurysm of heart |
|  | I25.4 | Coronary artery aneurysm and dissection |
|  | I25.5 | Ischaemic cardiomyopathy |
|  | I25.6 | Silent myocardial ischaemia |
|  | I25.8 | Other forms of chronic ischaemic heart disease |
|  | I25.9 | Chronic ischaemic heart disease, unspecified |

Supplement Table 2. Revascularization procedure codes used for defining coronary heart disease

| Procedure | Procedure Code | Description |
| --- | --- | --- |
| Coronary artery bypass grafting (CABG) | O1641 | Simple Aorta-Coronary Vascular Bypass Operation (Artery), 1 site |
|  | O1642 | Simple Aorta-Coronary Vascular Bypass Operation (Artery), 2 site or more |
|  | O1647 | Complex Aorta-Coronary Vascular Bypass Operation (Artery) |
|  | OA641 | Simple Off Pump CABG Aorta-Coronary Vascular Bypass Operation (Artery), 1 site |
|  | OA642 | Simple Off Pump CABG Aorta-Coronary Vascular Bypass Operation (Artery), 2 site or more |
|  | OA647 | Complex Off Pump CABG Aorta-Coronary Vascular Bypass Operation (Artery) |
| Percutaneous coronary intervention (PCI) | M6551 | Percutaneous Transluminal Coronary Angioplasty, single vessel |
|  | M6552 | Percutaneous Transluminal Coronary Angioplasty, additional vessel |
|  | M6561 | Percutaneous Trans-catheter Placement of Intracoronary Stent, single vessel |
|  | M6562 | Percutaneous Trans-catheter Placement of Intracoronary Stent, additional vessel |
|  | M6563 | Percutaneous Trans-catheter Placement of Intracoronary Stent with angioplasty or atherectomy, single vessel |
|  | M6564 | Percutaneous Trans-catheter Placement of Intracoronary Stent with angioplasty or atherectomy, additional vessel |
|  | M6571 | Percutaneous Transluminal Coronary Atherectomy, single vessel |
|  | M6572 | Percutaneous Transluminal Coronary Atherectomy, additional vessel |
